# Hypotensive and Antihypertensive Properties and Safety for Use of *Annona muricata* and *Persea americana* and Their Combination Products

**DOI:** 10.1155/2020/8833828

**Published:** 2020-12-10

**Authors:** Authentia Sokpe, Merlin L. K. Mensah, George A. Koffuor, Kwesi P. Thomford, Richmond Arthur, Yakubu Jibira, Michael K. Baah, Bright Adedi, Hope Y. Agbemenyah

**Affiliations:** ^1^Department of Pharmacognosy, Faculty of Pharmacy and Pharmaceutical Sciences, KNUST, Kumasi, Ghana; ^2^Department of Herbal Medicine, Faculty of Pharmacy and Pharmaceutical Sciences, KNUST, Kumasi, Ghana; ^3^Department of Pharmacology, Faculty of Pharmacy and Pharmaceutical Sciences, KNUST, Kumasi, Ghana; ^4^Department of Pharmacognosy and Herbal Medicine, University of Cape Coast, Cape Coast, Ghana; ^5^Department of Pharmacology and Toxicology, Centre for Plant Medicine Research, Mampong-Akuapem, Ghana; ^6^Department of Anatomy and Physiology, University of Health and Allied Sciences, Ho, Ghana

## Abstract

**Introduction:**

In the management of hypertension (a cardiovascular disease and the leading metabolic risk factor in noncommunicable diseases) with herbal medicines, efficacy and safety are of uttermost concern. This study sought to establish hypotensive, antihypertensive, drug interaction, and safety for use of the aqueous leaf extracts of *Annona muricata* (AME), *Persea americana* (PAE), or their combination products (CAPE). *Methodology*. Systolic and diastolic blood pressure (SBP and DBP), mean arterial blood pressure (MAP), and heart rate (HR) were measured in normotensive Sprague-Dawley rats treated with 50–150 mg/kg of AME, PAE, or CAPE to establish a hypotensive effect. “Combination index” was calculated to establish interaction between AME and PAE. The antihypertensive effect of CAPE was established by measuring SBP, DBP, MAP, and HR in ethanol-sucrose- and epinephrine-induced hypertension. Full blood count, liver and kidney function tests, and urinalysis were determined in ethanol/sucrose-induced hypertension to establish safety for use.

**Results:**

AME, PAE, and CAPE significantly (*p* ≤ 0.001) decreased BP in both normotensive and hypertensive animals. Effects of CAPE 1, CAPE 2, and CAPE 3 were synergistic (combination indices of 0.65 ± 0.07, 0.76 ± 0.09, and 0.87 ± 0.07, respectively). There was a significant decrease (*p* ≤ 0.01 − 0.001) in SBP and MAP with 100 mg/kg CAPE 1 and 75 mg/kg CAPE 2 treatment in hypertension as well as with nifedipine (*p* ≤ 0.001) treatment. Epinephrine-induced hypertension in anesthetized cats was significantly and dose-dependently inhibited (*p* < 0.05 − 0.001) by 25–100 mg/ml CAPE 1 and 37.5–75 mg/ml CAPE 2. CAPE administration had no deleterious effect (*p* > 0.05) on full blood count, liver and kidney function, and urine composition in hypertensive rats.

**Conclusion:**

The aqueous leaf extracts of *Annona muricata, Persea americana*, and their combination products possess antihypertensive properties, with combination products showing synergism and safety with use.

## 1. Introduction

Hypertension is a public health issue, a major contributor to cardiovascular diseases, and the leading metabolic risk factor in noncommunicable diseases. Data from the World Health Organization [1] revealed that 1.13 billion adults were affected by hypertension in 2015. Despite advances in the field of hypertension management, this figure is estimated to rise to over 1.5 billion adults by 2025 [2, 3]. In the provision of antihypertensive drugs, pharmaceutical industries have made giant strides in the development of novel synthetic medicines like thiazide diuretics, angiotensin-converting enzyme inhibitors (ACEI), angiotensin receptor blockers (ARBs), beta-blockers, and calcium channel blockers (CCB). However, these synthetic moieties have come with their own challenges [4, 5]. Many of these medicines though effective in managing hypertension are plagued with side effects for long-term users. Example can be given of the loop diuretics that may produce myalgia, hypokalaemia, and hyperglycaemia [6], the beta-blockers that may come with insomnia and asthmatic symptoms, the CCB that may cause palpitation, headache, constipation, dizziness, and pedal oedema, and the ACEI that may produce the chronic hacking dry cough, lung cancer, and kidney damage in its users [7, 8]. These factors have become the main drivers influencing the increased usage of herbal medicines by hypertensive patients [9–13].

Despite the popularity and dependency on herbal medicines, there is limited data on their safety and efficacy, and this is a topic of global importance. Many herbal medicines on the market are sold as food supplements due to the lack of preclinical and clinical data to substantiate their claims. More so, the assumption that single herb preparations can fit all is not sustainable since drugs whether botanical, biological, or chemical have inherent limitations if it focuses on only single target. It is important to address polygenic diseases from a syndrome-related metabolic cascade so that holistic management can be effectively achieved. Studies have also shown that plants when combined produce greater therapeutic effect at lower doses than the single herbal preparations [13–15]. This interaction is achieved through pharmacokinetic or pharmacodynamics mechanism.

*Persea americana,* commonly called avocado pear or alligator pear, is a fruit bearing tree and belongs to the family Lauraceae [16]. In Ghana, it is known as “*pεa* or *peya*” in Ewe, Ga, Fante, and Twi [17, 18]. The hypotensive and antihypertensive effects of *P. americana* leaf extract in normotensive and hypertensive Dahl salt-sensitive rats, respectively, have been reported by Ojewole et al. [19] and Owolabi et al. [20]. *Annona muricata* L. commonly known as soursop, prickly custard, or graviola, a naturally occurring evergreen fruit bearing tree, belongs to the family Annonaceae [21, 22]. In Ghana, the local names include *yevu-nyiklε* or *vo* (Ewe), *apere* (Fante), and *aprε* (Twi) [23]. The hypotensive effect of the aqueous leaf extract of *A. muricata* was reported by Nwokocha et al. [24] in Sprague-Dawley rats. This study therefore sought to establish activity, a possible drug interaction, and the safety for use of the aqueous leaf extracts of *A. muricata* and *P. americana* and their combination products in the management of hypertension.

## 2. Methodology

### 2.1. Ethical Consideration

The Committee on Animal Research, Publication and Ethics (CARPE) of the Department of Pharmacology, Faculty of Pharmacy and Pharmaceutical Sciences, KNUST, Ghana, approved this study from an ethical point of view (Ethics Reference Number: FPPS/PCOL/008/2017).

### 2.2. Plant Collection and Authentication

The matured leaves of *Annona muricata* and *Persea americana* were obtained from Titrinu-Ho (VA-2653-9626) and Kpoeta Ashanti (VH-0147-5075), respectively, in the Volta Region of Ghana, in November 2017. The plant materials were authenticated by Dr. George Henry Sam of the Department of Herbal Medicine, Faculty of Pharmacy and Pharmaceutical Sciences, KNUST, Kumasi, Ghana. Voucher specimens have been deposited at the Herbarium of the Department, coded *Annona muricata* (KNUST/HM1/2019/L009) and *Persea americana* (KNUST/HM1/2014/L003 (77)).

### 2.3. Preparation of Plant Extracts and Combination Products

The fresh leaves of *A. muricata* were washed under running tap water to get rid of foreign matter and dried on a wire mesh under the shade at ambient temperature (24–35°C) for ten (10) days. The dried leaves were milled into a fine powder with a general-purpose electric blender (Vitamix 5200, Canada). The fresh leaves of *P. americana* were also processed as described above. A weight of 0.7 kg *A. muricata* and 2.5 kg *P. americana* was infused separately using hot water for 30 minutes and filtered. The filtrates were lyophilized using a vacuum freeze dryer (YK-118-50, Taiwan) at the Council for Scientific and Industrial Research, Fumesua, Kumasi, Ghana. The lyophilized powders obtained were labeled as aqueous leaf extract of *A. muricata* (AME) and aqueous leaf extract of *P. americana* (PAE) and kept in an airtight glass container with a desiccator until required for use. A combination product of AME : PAE (CAPE) in the ratios 1 : 1, 1 : 2, and 1 : 3 were prepared and labeled CAPE 1, CAPE 2, and CAPE 3, respectively, for use in this study.

### 2.4. Experimental Animal and Husbandry

Healthy adult Sprague-Dawley rats 12 weeks old weighing 140–240 g obtained from Centre for Plant Medicine Research (CPMR), Akuapem-Mampong in the Eastern Region of Ghana, were used in this study. Animals were housed in stainless steel cages (34 × 47 × 18 cm) lined with wood shavings. The rats were fed on standard rat chow from Agricare Ltd, Kumasi, Ghana, and water *ad libitum*. The animals were kept in a well-ventilated room within a temperature range of 25–30°C and ambient light and dark cycle of 10–16 hours, respectively.

Healthy adult cats (2 years old, weighing 1.4–2.5 kg) obtained from the Animal Facility of Department of Pharmacology, Kwame Nkrumah University of Science and Technology, were used in the epinephrine-induced hypertension experimental model. They were fed on a normal diet (cornmeal mixed with dried fish) and water *ad libitum.* The animals were kept in a well-ventilated room within a temperature range of 25–30°C and ambient light and dark cycle of 10–16 hours, respectively. All animals were treated in accordance with the National Institute of Health Guidelines for the Care and Use of Laboratory Animals (Directive 2010/63/EU; Animal Care and Use Committee, 1998).

### 2.5. Hypotensive and Interactive Effects

The hypotensive effects of AME, PAE, and CAPE and possible drug interaction between AME and PAE were investigated. To start up, the systolic and diastolic BP and heart rate of grouped (I-VII) normotensive rats (*n* = 6) were initially recorded using the noninvasive tail-cuff BP apparatus (UGO Basile 58500, Italy). Groups I–III were then treated with 50, 100, and 150 mg/kg of AME, respectively, and Groups IV–VI with 50, 100, and 150 mg/kg of PAE, respectively, by oral gavage as single doses. Group VII (control) was treated with distilled water and kept under the same experimental conditions. The systolic and diastolic BP and heart rate were then measured at 4, 8, and 24 hours after treatment.

Combinations of AME and PAE (CAPE) in the ratios 1 : 1, 1 : 2, and 1 : 3 of CAPE 1 (100 mg/kg), CAPE 2 (150 mg/kg), and CAPE 3 (200 mg/kg) were studied as described above. Chou–Talalay method for drug combination (based on the median-effect equation) [25] was employed to establish the “combination index (CI).” For each product combination, the IC_50_ (experimental), CI, and its associated fraction affected (Fa) were evaluated by a quantitative diagnostic plot (Fa-CI) analysis of the log dose-response curve. The CI was obtained from the following formulas:

(1) $$\begin{array}{c} \frac{\text{Fa}}{\text{Fu}}={\left[\frac{D}{Dm}\right]}^{m}, \end{array}$$

(2) $$\begin{array}{c} \log\left[\frac{\text{Fa}}{\text{Fu}}\right]=m\left[\log\;D\right]-m\left[\log\;Dm\right], \end{array}$$

(3) $$\begin{array}{c} \text{CI}=\sum_{j=1}^{n}\left[\frac{D}{Dx}\right], \end{array}$$

where Fa = fraction affected, Fu = fraction unaffected, *D* = dose required to produce Fa, *Dm* = the median dose effect (IC_50_), *m* = dynamic order (sigmoidicity), and *Dx* = the dose of each drug alone that exerts *X* % inhibition. *P* values for statistical significance were set at 0.05. Synergism is achieved if CI < 1 [25] (Table 1). The mean arterial blood pressure (MAP) was then calculated from the systolic blood pressure (SBP) and diastolic blood pressure (DBP) as follows:

(4) $$\begin{array}{c} \text{MAP}=\frac{1}{3}\left(\text{SBP}+2\text{DBP}\right). \end{array}$$

### 2.6. Antihypertensive Effect

#### 2.6.1. Ethanol-Sucrose-Induced Hypertension

The ethanol-sucrose-induced hypertension protocol as described by Vasdev et al. [26] and Dzeufiet et al. [27] in adult Sprague-Dawley rats was used in this study, with slight modification. The initial systolic and diastolic blood pressure (SBP and DBP) and heart rate (HR) before induction of hypertension were noted. Hypertension was then induced in the rats by giving 3.2 ml/kg of 40 %v/v ethanol by oral gavage and 10% w/v sucrose solution as drinking water, measuring the SBP and DBP weekly until hypertension (SBP > 140 mmHg; DBP > 90 mmHg) was induced after 6 weeks. Hypertensive animals were then put into 6 groups labeled I-V (*n* = 6) and maintained on ethanol-sucrose. CAPE 1 (50 and 100 mg/kg) and CAPE 2 (75 mg/kg) were then administered orally in Groups I–III, respectively. A 0.5 mg/kg dose of nifedipine (reference drug) was administered to Group IV. Group V (negative control) was treated with normal saline after induction. Treatment of hypertensive rats was done for two (2) weeks with weekly SBP, DBP, and HR measurements.

#### 2.6.2. Epinephrine-Induced Hypertension

The adult cat was anesthetized by intramuscular administration of a mixture of 5 mg/ml pentobarbitone and 50 mg/ml *α*-chloralose. The femoral vein and the carotid artery were carefully exposed by shaving off the hair in the area and making an incision in the groin and left side of the neck, respectively. A 20 G cannula was inserted into the femoral vein through which normal saline and the drugs were to be administered. An 18 G cannula was also inserted into the carotid artery which was then connected to a pressure transducer (P23Gb, Statham Instruments, Inc.) through a three-valve catheter. This also served as a passage to introduce heparinized saline to prevent blood coagulation. Care was taken to allow the cat to breathe well. The body temperature was maintained at room temperature [28, 29]. The initial BP reading was recorded on the pressure transducer. Force of contraction and heart rate were also recorded on kymograph (model 50–822, Harvard Apparatus Ltd., Kent, England). CAPE 1 was administered at concentrations of 15, 25, 50, and 100 mg/ml. In another experiment, CAPE 2 was administered at concentrations of 22.5, 37.5, and 75 mg/ml to determine the effect on the normotensive cat. At each time, the BP, force of contraction, and heart rate were recorded. To determine the effect of CAPE 1 and CAPE 2 on the hypertensive cat, the products at the same concentrations were coadministered with 20 *μ*g/ml epinephrine. Nifedipine (75 *μ*g/ml) was used as the reference antihypertensive drug. Each experiment was done in triplicate [29].

#### 2.6.3. Safety Assessment of CAPE in Hypertension

At the end of treatment in the ethanol-sucrose-induced hypertensive rat model, blood samples from rats in the various treatment groups were obtained from the jugular vein using standard protocols [30, 31] into labeled ethylenediaminetetraacetate (EDTA) tubes (Mediplus, Mumbai) for hematological profiling and gel separation tubes (Mediplus, Mumbai) for biochemical analysis. Full blood count was done at Noguchi Memorial Institute for Medical Research using an automated haematology analyser (SYSMEX KX-21N, USA) whilst liver and kidney function tests were performed on the samples at the ChemPath Laboratory, Mampong-Akuapem, using a fully automated chemistry analyser (PENTRA C200, Belgium).

#### 2.6.4. Urine Analysis

Semiquantitative urinalysis was done on freshly obtained urine sample from the urinary bladder of rats using the dipstick (Insight Urinalysis Reagent Strips, Veltlab Ltd.) following the protocol as stated by the manufacturer. Parameters measured include bilirubin, blood, glucose, leucocytes, ketones, nitrites, protein, specific gravity, pH, and urobilinogen.

### 2.7. Statistical Analysis

Values for parameters measured were expressed as means ± SEM. Significant differences in treatments were analysed using one-way analysis of variance (ANOVA). Multiple comparisons between groups were done using Dunnett's *post hoc* test. All statistical analyses were carried out with MS Excel (Microsoft Office, 2016) and GraphPad Prism 6 (GraphPad software, San Diego, California, USA). Values are considered to be statistically significant at *p* value less than or equal to 0.05.

## 3. Results

### 3.1. Hypotensive and Drug Interactive Effects

**Figure 1:**
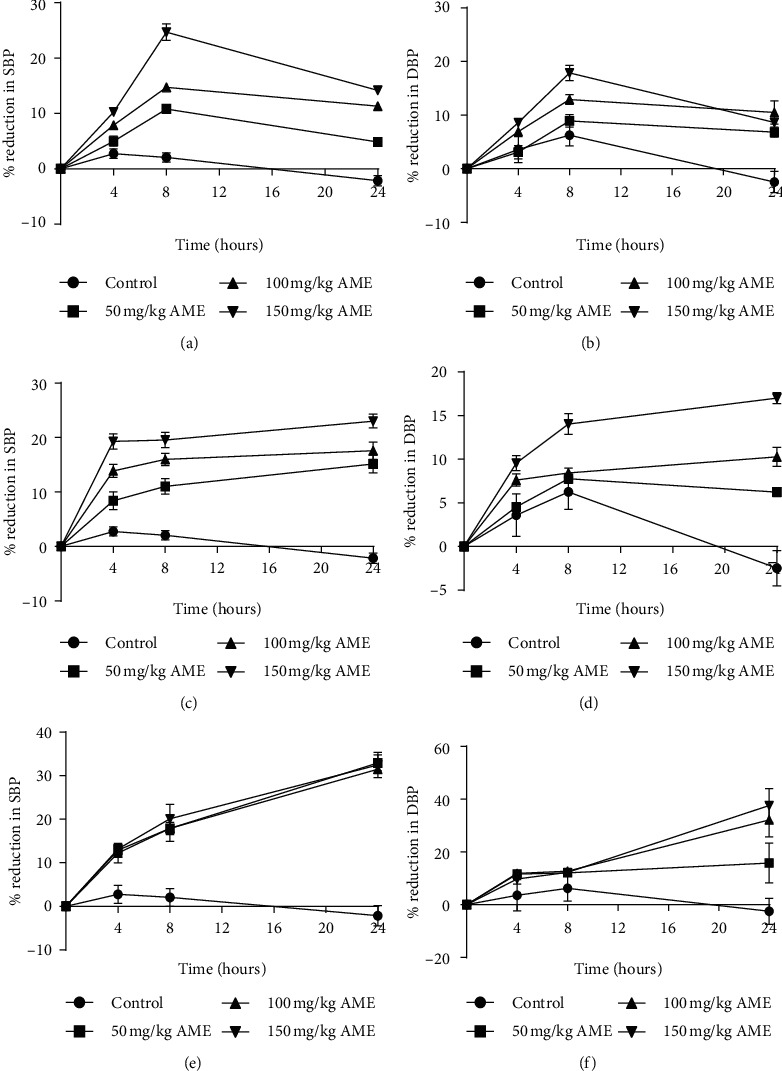
Time course of effect of aqueous extracts of *A. muricata* (a, b) (AME), (c, d) *P. americana* (PAE) and their combination products (e, f) (CAPE) on systolic and diastolic BP in normotensive SD rat. Values are plotted as mean ± SEM (*n* = 6). CAPE 1, CAPE 2, and CAPE 3 are combinations of AME : PAE in the ratios 1 : 1, 1 : 2, and 1 : 3, respectively.

**Figure 2:**
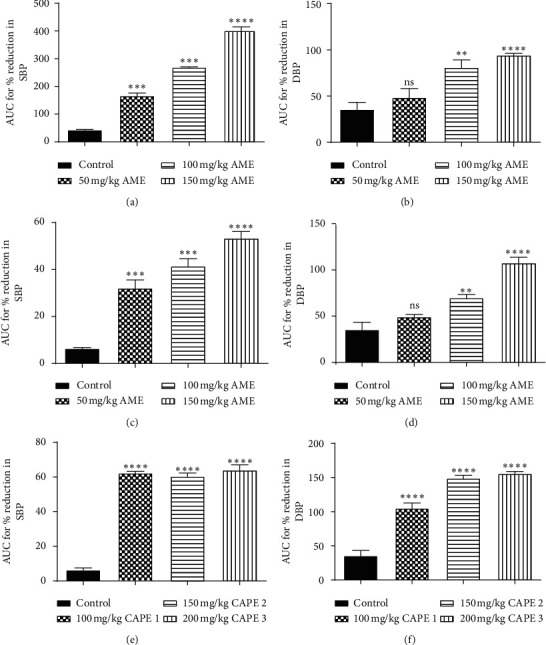
Plots for area under the curve (AUC) for the time course of effect of (a, b) AME, (c, d) PAE, and (e, f) CAPE on systolic and diastolic BP in normotensive rats. Values are plotted as mean ± SEM (*n* = 6). ^*∗∗∗*^*p* ≤ 0.001, ^*∗∗*^*p* ≤ 0.01, ns: *p* > 0.05; compared to the control (one-way ANOVA with Dunnett's multiple comparisons post hoc test). CAPE 1, CAPE 2, and CAPE 3 are combinations of AME and PAE in the ratios 1 : 1, 1 : 2, and 1 : 3, respectively.

**Figure 3:**
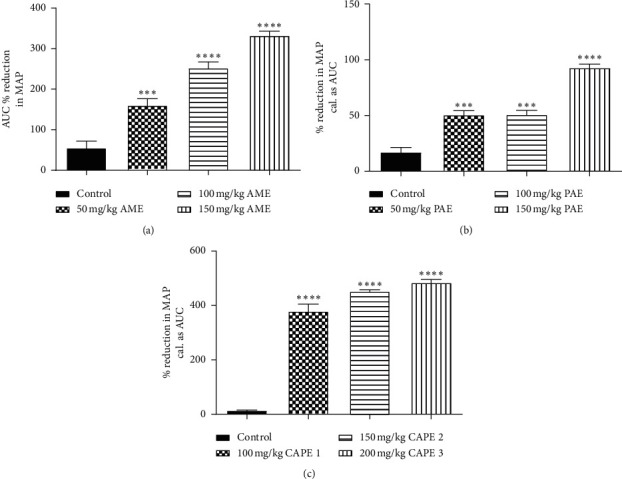
Percentage reduction in mean arterial pressure (MAP) caused by (a) AME, (b) PAE, and (c) CAPE administration to SD rats within 24 hours. Values are plotted as mean ± SEM (*n* = 6). ^*∗∗∗*^*p* ≤ 0.001, compared to the control (one-way ANOVA with Dunnett's multiple comparison post hoc test). CAPE 1, CAPE 2, and CAPE 3 are combinations of AME and PAE in the ratios 1 : 1, 1 : 2, and 1 : 3, respectively.

AME and PAE as well as CAPE 1, CAPE 2, and CAPE 3 dose-dependently and significantly decreased (*p* ≤ 0.01 − 0.001) SBP, DBP, and MAP in normotensive rats. The effect of AME peaked at the 8th hour posttreatment and waned towards the 24th hour. PAE had a more sustained effect over the 24-hour period (Figures 1–3). AME and PAE had no effect (*p* > 0.05) on heart rate; however, CAPE 1, CAPE 2, and CAPE 3 caused a significant reduction (*p* ≤ 0.001) of heart rate by the 4th hour, with the effect waning by the 8th hour (Table 2). For drug interaction, CAPE 1, CAPE 2, and CAPE 3 caused synergistic effect recording CIs of 0.65 ± 0.07, 0.76 ± 0.09, and 0.87 ± 0.07, respectively.

### 3.2. Antihypertensive Effect

#### 3.2.1. Ethanol/Sucrose-Induced Model

**Figure 4:**
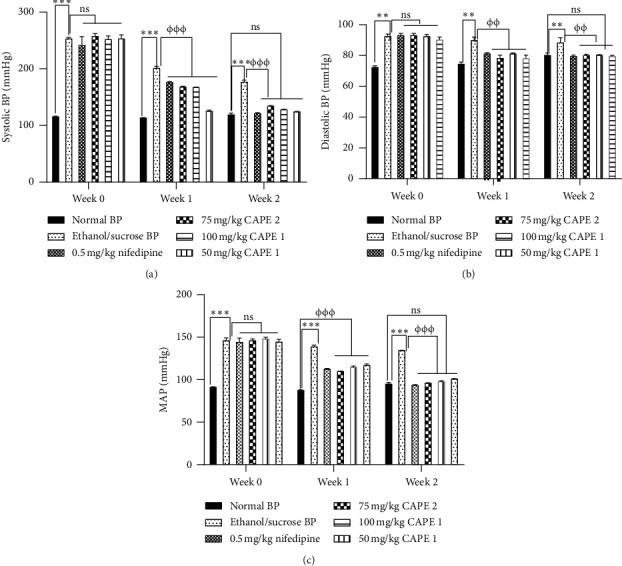
Effect of various concentrations of CAPE 1, CAPE 2, and nifedipine on (a) systolic blood pressure (SBP), (b) diastolic pressure (DBP), and (c) mean arterial pressure (MAP) in ethanol/sucrose-induced hypertension. Values are plotted as mean ± SEM. ^*∗∗∗*^*p* ≤ 0.001 compared to the normal control group and also ^*φφφ*^*p* ≤ 0.001 compared to the negative control group; one-way ANOVA with Dunnett's multiple comparison post hoc test.

Administration of ethanol and sucrose solution significantly (*p* ≤ 0.001) induced hypertension in Sprague-Dawley rats by the elevation of SBP, DBP, and MAP compared to that for the normal control group which had minimal variations in the BP readings throughout the experimental period. The elevated SBP, DBP, and MAP in all treatment groups reduced significantly (*p* ≤ 0.001) over the period compared to the negative control. There was no difference (*p* > 0.05) in the measured parameters in week 2 of treatment as compared to the normal control group (Figure 4).

#### 3.2.2. Epinephrine-Induced Hypertension

**Figure 5:**
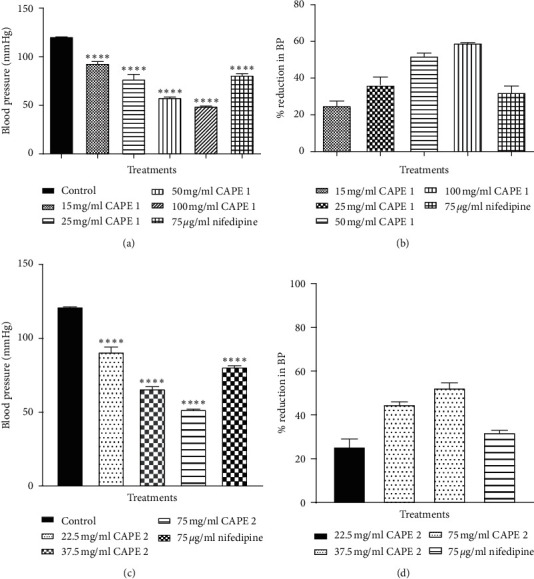
Effect of (a, c) various concentrations of CAPE 1 and CAPE 2 on the blood pressure of normotensive cats, (b, d) showing percentage reduction in BP. Values are plotted as mean + SEM (*n* = 3). ^*∗*^*p* ≤ 0.05, ^*∗∗*^*p* ≤ 0.01; ^*∗∗∗*^*p* ≤ 0.001 as compared to the control group; one-way ANOVA with Dunnett's multiple comparison post hoc test.

All concentrations of CAPE 1 (15–100 mg/ml) and CAPE 2 (22.5–75 mg/ml) caused a significant reduction (*p* ≤ 0.05 − 0.001) in blood pressure in normotensive cats similar to that caused by 75 *µ*g/ml nifedipine (Figure 5).

**Figure 6:**
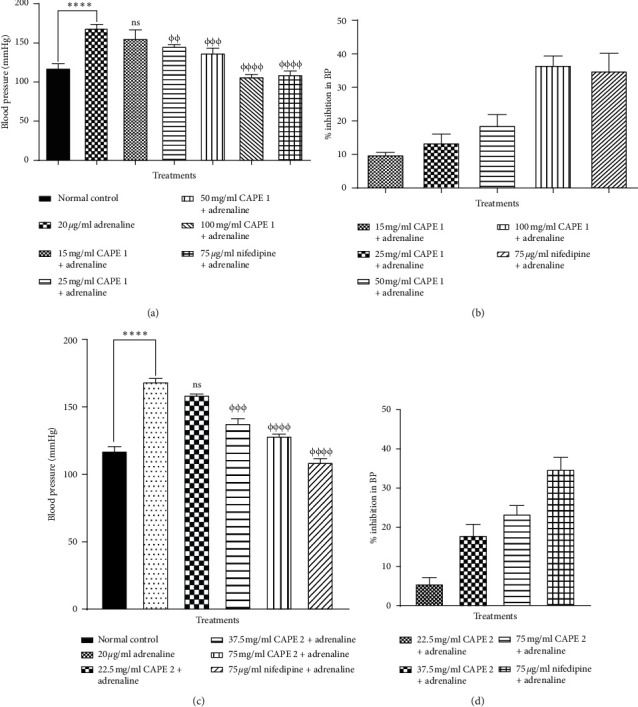
Effect of (a, c) various concentrations of CAPE 1 and CAPE 2 on the blood pressure inhibition of epinephrine-induced hypertensive cats, (b, d) showing percentage inhibition of BP. Values are plotted as mean ± SEM (*n* = 3). ^*∗∗∗∗*^*p* ≤ 0.001 as compared to the control group, ^*φφφ*^*p* ≤ 0.01; ^*φφφφ*^*p* ≤ 0.001 as compared to the hypertensive control; one-way ANOVA with Dunnett's multiple comparison post hoc test.

Epinephrine caused a significant increase (*p* ≤ 0.001) in blood pressure cat, but this effect was dose-dependently and significantly inhibited by all concentrations of CAPE 1 and CAPE 2. Comparison of the inhibitory effect of the combined extract with that of nifedipine revealed that 100 mg/ml CAPE 1 and 75 mg/ml CAPE 2 had similar effects (Figure 6).

### 3.3. Safety Assessment

Safety for use of the combination products of AME and PAE in hypertension was assessed, employing the ethanol/sucrose-induced hypertensive model. Study results indicated no detrimental effect on hematological profile (Table 3). Except for total bilirubin (TBIL) which was significantly reduced (*p* ≤ 0.001) at all dose levels of CAPE 1 and CAPE 2, liver function parameters were not affected (*p* > 0.05), i.e., comparable to the normal control (Table 4). CAPE administration also did not affect kidney function parameters. Urinalysis revealed the reversal of proteinuria caused by the induction of hypertension in the CAPE- and nifedipine-treated rats (Table 5).

## 4. Discussion

This study sought to establish antihypertensive activity and safety for use of aqueous extracts of *Annona muricata* (AME) and *Persea americana* (PAE) and their combination products (CAPE); hence, the study involved the measurement of systolic and diastolic blood pressure as well as force and rate of myocardial contraction. Blood pressure is the product of cardiac output and total peripheral resistance. A persistent increase in SBP/DP indicates hypertension [32]. Treatment of normotensive rats with AME, PAE, and CAPE 1, CAPE 2, and CAPE 3 resulted in significantly decreased SBP, DBP, and hence MAP, indicating hypotensive effect. This possibly suggests that the extracts and their combination product contain phytochemicals which possess the ability to possibly cause reduction in cardiac output and/or total peripheral resistance. A decrease in cardiac output results in reduction in the total volume of blood flowing into peripheral blood vessels [33] and hence a reduction in the pressure exerted on the walls of the vessel [33]. Dilatation of the peripheral blood vessel would result in a fall in blood pressure [34] as resistant to blood flow decreases [34]. This will then constitute a fall in blood pressure, i.e., hypotension [34].

Although AME and PAE had no significant effect on heart rate, the combination products caused a significant reduction in heart rate by the 4th hour, suggesting negative chronotopy and hence a reduction in cardiac output [33] and a fall in BP. This finding could also suggest a possible effect by phytochemicals on the rate of impulse firing by the pacemaker node in the myocardium. The pacemaker node initiates action potential leading to the propagation of electrical impulse through the electrical conduction system of the heart causing myocardial contraction [35, 36] with an overall effect on the blood pressure.

For only the combination products to elicit a significant effect on heart rate, there is reason to suggest possible drug interaction. This interaction was confirmed by the “combination index (CI)” obtained in this study, as mild to severe synergism between AME and PAE as per the Chou–Talalay method for drug combination [25]. Synergy is the desired effect sought to achieve a greater effect than that attained by any drug singly [37]. A synergistic product would therefore be of much preference as an antihypertensive product as its efficacy would be much more enhanced, and toxicity effects reduced (due in part to lower doses of drug administered).

To further confirm the significance of the combination product of AME and PAE in hypertension management, the ethanol/sucrose-induced [26, 27] and epinephrine-induced hypertensive models [28, 29] were used in this study. Administration of ethanol over a significant period of time stimulates the release of endothelin 1 and 2 from vascular endothelium [38] and the release of angiotensin 2 from the renin-angiotensin-aldosterone system (RAAS). These endogenous chemicals are potent vasoconstrictors [39], thus causing vasoconstriction of peripheral vessels [39] and hence an increase in total peripheral resistance [40, 41]. Furthermore, chronic administration of sucrose stimulates peripheral sympathetic activity and also reduces the baroreceptor reflex with a resultant increase in blood pressure [39, 42]. The elevated SBP, DBP, and MAP reduced significantly with treatments indicating antihypertensive effect. The combination products of the extracts may have attained their effect by possibly inhibiting the renin-angiotensin-aldosterone system. Treatment of normotensive cats with the combination products also revealed hypotensive effect, confirming earlier observation in SD rats.

Epinephrine administration caused a significant increase in the blood pressure of the anesthetized cat. Epinephrine acts on a presynaptic beta-receptor on sympathetic nerve endings which indirectly leads to a sustained increase in the neuronal release of norepinephrine [43]. Epinephrine-induced BP was significantly inhibited by all concentrations of CAPE 1 and CAPE 2, in a manner similar to that of nifedipine. Nifedipine reduces blood pressure by inhibiting the L-type voltage-sensitive calcium channels in vascular smooth muscles and myocardial cells [44]. The similar effect produced by the products and nifedipine may possibly suggest a calcium channel blocking mechanism on the myocardium and hence a reduction in force and rate of myocardial contraction (an effect noticed earlier in this study) [45].

Several research findings have revealed the presence of alkaloids, tannins, flavonoids, essential oils, and phenolic compounds in *Annona muricata* and *Persea americana* [46, 47] which could have contributed to their antihypertensive effect. For instance, Dias et al. [48] have reported that reticuline reduced blood pressure through voltage-dependent Ca^2+^ channel blockade and/or inhibition of Ca^2+^ release from norepinephrine-sensitive intracellular stores. Reticuline, an alkaloid, is one of the components of *A. muricata* leaves [49, 50]. Also, some essential oils such as beta-caryophyllene present in other *P. americana* have been reported to exhibit hypotensive and vasodilator activities [51].

Complications in hypertension involve hematological, liver, and kidney disorders. Safety assessment of the combination products in hypertension management in experimental animals revealed no detrimental effect on hematological profile and liver and kidney function parameters. Urinalysis revealed the reversal of proteinuria caused by the induction of hypertension. Some herbal medicines can affect the osmotic fragility of blood cells causing cell lysis, resulting in decreased RBCs, haemoglobin, WBCs, PLTs which ends up with anemia, leucocytopenia, and thrombocytopenia [52]. These hematological disorders have several morbidity effects and could eventually result in mortality. Hemolysis of RBCs results in increased bilirubin levels in the blood causing cholestasis and jaundice, with eventual appearance in urine, as well as increased urobilinogen levels in the urine [53]. Others also decrease erythropoiesis resulting in decreased RBC levels and decreased haemoglobin levels [52]. The fact that the products have no effect on hematological profile implies that the product may not have affected hematopoiesis, and may not have hemolyzing tendencies, which augur well for therapeutic use.

Except for total bilirubin (TBIL) which was significantly reduced, liver function parameters were not affected by treatments. The liver is the main organ for drug metabolism and therefore prone to injury; the commonest enzymes employed as indicators of hepatocellular damage are the aminotransferase enzymes and alkaline phosphatase (ALP) [54]. Damage to the liver results in increased levels of these enzymes in the plasma and this is usually proportional to the extent of tissue damage [54]. The reduction of the total bilirubin on the administration of the product indicates a possible hepatoprotective effect [55] of CAPE which is preferred in hypertension management.

CAPE administration did not affect kidney function parameters, indicating that the product did not have any deleterious effect on kidney function. Elevated level of serum creatinine and urea would have indicated adverse effect on the kidneys [56].The “no adverse effect” on hematological profile, as well as on the liver and kidneys, was confirmed in the urinalysis results. Breakdown products in these organs/systems will always be excreted into urine, hence the importance of performing urinalysis. Induction of hypertension resulted in elevated levels of protein in the urine (proteinuria) [57]. This was however reversed after treatment with CAPE, confirming the antihypertensive property of the product and its safety for use in hypertension.

## 5. Conclusion

This study showed that aqueous extracts of *Annona muricata*, *Persea americana*, and their combination products have hypotensive and antihypertensive properties, with the combination products eliciting synergism on administration. The combination products are safe to use in hypertension, hence having a promising therapeutic advantage for prevention and treatment of hypertension in Ghana. It is recommended that chronic toxicity studies and possible mechanism of action of the combination products should further be studied and established.

## Figures and Tables

**Table 1:** Description of drug interactive effects analysed using the combination index method.

Range of combination index (CI)	Description
<1.0	Very strong synergism
0.10–0.30	Strong synergism
0.30–0.70	Synergism
0.70–0.85	Moderate synergism
0.85–0.90	Slight synergism
0.90–1.10	Nearly additive
1.10–1.20	Slight antagonism
1.20–1.45	Moderate antagonism
1.45–3.30	Antagonism
3.30–10	Strong antagonism
>10	Very strong antagonism

[25].

**Table 2:** Effect of AME, PAE, and CAPE on the heart rate of normotensive rats.

Time (h)		50 mg/kg	100 mg/kg	150 mg/kg
*AME*
0		400.17 ± 8.40	422.56 ± 14.14	432.67 ± 20.00
4		408.61 ± 12.71^ns^	399.06 ± 13.34^ns^	400.78 ± 13.58^ns^
8		408.39 ± 65.88^ns^	404.89 ± 14.19^ns^	410.67 ± 8.67^ns^
24		410.39 ± 18.00^ns^	423.50 ± 20.01^ns^	403.78 ± 7.72^ns^

*PAE*
0		415.00 ± 2.13	415.00 ± 2.13	415.28 ± 3.98
4		410.89 ± 4.82^ns^	415.56 ± 6.26^ns^	413.56 ± 2.70^ns^
8		407.67 ± 2.31^ns^	406.11 ± 3.16^ns^	414.89 ± 2.61^ns^
24		422.11 ± 4.42^ns^	439.33 ± 22.46^ns^	408.39 ± 6.59^ns^

*CAPE*		*CAPE 1*	*CAPE 2*	*CAPE 3*
0		424.44 ± 9.22	430.00 ± 7.00	418.78 ± 9.85
4		346.66 ± 5.33^*∗∗∗∗*^	339.00 ± 1.73^*∗∗∗∗*^	311.89 ± 3.11^*∗∗∗∗*^
8		413.89 ± 5.39^ns^	416.33 ± 11.18^ns^	407.44 ± 6.48^ns^
24		394.45 ± 14.89^ns^	423.89 ± 10.39^ns^	406.89 ± 11.50^ns^

Values are recorded as mean ± SEM, *n* = 6. Comparison of hours of treatment to the zero hour: ns: *p* > 0.05; ^*∗∗∗∗*^*p* ≤ 0.001; one-way ANOVA followed by Dunnett's multiple comparisons test.

**Table 3:** Effect of 50, 75, and 100 mg/kg CAPE and nifedipine on full blood count.

Parameter	Combined extract (mg/kg)			0.5 mg/kg nifedipine	Negative control	Normal control
	50 CAPE 1	75 CAPE 2	100 CAPE 1			
WBC (×10^3^/uL)	10.03 ± 1.25	9.08 ± 0.51	14.60 ± 0.32	11.48 ± 1.36	10.10 ± 0.79	12.60 ± 0.20
RBC (×10^6^/uL)	8.16 ± 0.30	7.77 ± 0.56	8.01 ± 0.14	8.02 ± 0.30	8.16 ± 0.17	7.70 ± 0.14
HGB (g/dL)	14.73 ± 0.86	13.96 ± 1.02	14.14 ± 0.17	14.30 ± 0.11	14.35 ± 0.16	13.95 ± 0.65
HCT (%)	45.03 ± 1.48	42.86 ± 3.63	44.66 ± 1.28	43.40 ± 1.58	44.98 ± 0.41	44.25 ± 0.95
MCV (fL)	55.22 ± 0.70	54.96 ± 1.14	55.72 ± 0.97	54.15 ± 0.59	55.23 ± 0.85	57.50 ± 0.20
MCH (pg)	18.02 ± 0.53	17.98 ± 0.36	17.68 ± 0.26	17.93 ± 0.58	17.63 ± 0.48	18.10 ± 0.50
MCHC (g/dL)	32.62 ± 0.58	32.74 ± 0.51	31.72 ± 0.65	33.08 ± 1.05	31.90 ± 0.41	31.50 ± 0.80
PLT (×10^3^/uL)	614.17 ± 90.97	802.20 ± 78.86	668.00 ± 77.60	700.25 ± 118.89	693.50 ± 92.47	635.00 ± 30.00
LYM%	65.40 ± 2.61	69.74 ± 3.01	65.64 ± 2.52	67.05 ± 1.88	66.05 ± 1.86	69.50 ± 0.30
LYM# (×10^3^/uL)	6.58 ± 0.92	6.30 ± 0.39	9.56 ± 0.33	7.75 ± 1.12	6.68 ± 0.57	8.75 ± 0.05
RDW_SD (fL)	29.08 ± 0.65	26.56 ± 3.39	30.48 ± 0.66	30.70 ± 1.19	29.13 ± 0.63	29.70 ± 1.40
RDW_CV (%)	14.73 ± 0.70	13.10 ± 0.97	14.10 ± 0.72	16.03 ± 1.95	13.35 ± 0.93	12.50 ± 1.00
PDW (fL)	10.17 ± 0.20	10.16 ± 0.61	10.40 ± 0.74	10.73 ± 0.59	9.78 ± 0.09	9.90 ± 0.30
MPV (fL)	7.75 ± 0.13	8.90 ± 0.93	7.86 ± 0.29	7.63 ± 0.17	7.80 ± 0.17	7.65 ± 0.05
P_LCR (%)	11.77 ± 0.76	13.68 ± 0.26	13.24 ± 1.73	12.00 ± 0.98	12.10 ± 0.90	11.90 ± 0.60

Values are expressed as mean ± SEM (*n* = 5). Statistical analysis indicated no significant difference (*p* > 0.05) (one-way ANOVA with Dunnett's post hoc test). WBC: white blood cell; RBC: red blood cell; HGB: haemoglobin; HCT: haematocrit; MCV: mean cell volume; MCH: mean cell haemoglobin; MCHC: mean cell haemoglobin concentration; PLT: platelet; LYM: lymphocyte; RDW: red cell distribution width; SD: standard deviation; CV: coefficient of variation; PDW: platelet distribution width; MPV: mean platelet volume; P_LCR: platelet large cell ratio.

**Table 4:** Effect of CAPE and nifedipine on liver and kidney function in ethanol/sucrose-induced hypertension in SD rats.

Parameter	Dose of combined product (mg/kg)			Nifedipine (0.5 mg/kg)	Negative control	Normal control
	50 CAPE 1	100 CAPE 1	75 CAPE 2			
ALP (U/L)	135.3 ± 7.88	152.9 ± 11.09	134.7 ± 31.23	—	149.5 ± 24.82	95.27 ± 4.54
Total protein (g/L)	63.62 ± 0.85	67.44 ± 1.13	66.86 ± 1.52	71.65 ± 1.55	66.76 ± 2.60	69.30 ± 0.80
Albumen (g/L)	31.80 ± 0.84	29.46 ± 0.63	32.28 ± 1.21	32.87 ± 0.90	31.38 ± 1.34	31.53 ± 0.57
AST (U/L)	158.9 ± 14.58	155.5 ± 16.47	145.3 ± 10.54	168.7 ± 4.615	148.5 ± 15.45	152.0 ± 11.69
ALT (U/L)	41.20 ± 1.62	41.33 ± 2.40	38.50 ± 4.11	31.75 ± 1.63	40.13 ± 4.72	31.17 ± 2.69
GGT (U/L)	6.0 ± 0.0	6.0 ± 0.0	6.0 ± 0.0	6.0 ± 0.0	6.0 ± 0.0	6.0 ± 0.0
TBIL (*µ*mol/L)	3.94 ± 0.25^*∗∗∗*^	4.90 ± 0.41^*∗∗∗*^	4.54 ± 0.22^*∗∗∗*^	9.18 ± 0.14	9.74 ± 0.39	11.77 ± 0.29
Urea (mmol/L)	6.91 ± 0.58	6.99 ± 0.40	7.08 ± 0.47	—	7.78 ± 0.32	8.54 ± 0.33
Creatinine (*µ*mol/L)	88.44 ± 2.57	88.13 ± 2.77	96.02 ± 3.13	—	83.84 ± 3.77	98.82 ± 2.00
Na (mmol/L)	143.6 ± 0.39	143.7 ± 0.70	144.4 ± 0.55	142.9 ± 0.47	143.3 ± 1.07	143.2 ± 0.69
K (mmol/L)	4.92 ± 0.18	5.43 ± 0.07	5.21 ± 0.18	5.86 ± 0.29	5.65 ± 0.43	5.72 ± 0.14
Cl (mmol/L)	102.9 ± 0.64	104.0 ± 0.76	105.1 ± 0.76	100.2 ± 0.91	102.4 ± 0.39	101.6 ± 1.57

Values are expressed as mean ± SEM (*n* = 5). ^*∗∗∗*^*p* ≤ 0.001 as compared to the normal control (one-way ANOVA with Dunnett's multiple comparison post hoc test). ALP: alkaline phosphatase; T: total; AST: aspartate aminotransferase; ALT: alanine aminotransferase; GGT- *γ*: glutamyltransferase; Na sodium; K: potassium; Cl: chloride.

**Table 5:** Effect of 50, 75, and 100 mg/kg CAPE and nifedipine on urine content in ethanol/sucrose-induced hypertension in SD rats.

Parameter	Combined extract (mg/kg)			0.5 mg/kg nifedipine	HC	NC
	50 CAPE 1	75 CAPE 2	100 CAPE 1			
Leukocyte	Trace	Trace	Trace	Trace	Trace	Trace
Urobilinogen	Trace	Trace	Trace	Trace	Trace	Trace
Bilirubin	Negative	Negative	Negative	Negative	Negative	Negative
Blood	Negative	Negative	Negative	Negative	Negative	Negative
Nitrite	Negative	Negative	Negative	Negative	Negative	Negative
Ph	8.00 ± 0.13	8.50 ± 0.00	7.83 ± 0.31	7.58 ± 0.30	7.75 ± 0.25	7.13 ± 0.13
SG	1.01 ± 0.00	1.01 ± 0.00	1.02 ± 0.00	1.02 ± 0.00	1.01 ± 0.00	1.08 ± 0.06
Protein	++	++	++	++	+++	++
Glucose	Negative	Negative	Negative	Negative	Negative	Negative
Ketones	Negative	Negative	Negative	Negative	Negative	Negative

Values are expressed as mean ± SEM (*n* = 5). SG: specific gravity; HC: negative control; NC: normal control.

## Data Availability

All data generated or analysed during this study are included in this published article (and its supplementary information files). However, the datasets used and/or analysed during the current study are available from the corresponding author on reasonable request.
